# Role of Feed Processing on Gut Health and Function in Pigs and Poultry: Conundrum of Optimal Particle Size and Hydrothermal Regimens

**DOI:** 10.3389/fvets.2019.00019

**Published:** 2019-02-19

**Authors:** Elijah G. Kiarie, Alisha Mills

**Affiliations:** Department of Animal Biosciences, University of Guelph, Guelph, ON, Canada

**Keywords:** antibiotic-free feeding programs, gut health and function, exogenous feed enzymes, feed particle size and hydrothermal processing, pigs, poultry, nutrition

## Abstract

The aim is to give an overview of available literature data on the role of feed processing on gut health and function with specific focus on particle size and hydrothermal processing. In addition, influence of feed processing on efficacy of exogenous feed enzymes will be discussed. The current feed processing technologies are such that ingredient choices and diet form are refined to improve feed intake and nutrient utilization efficiency. Finer feed particle size enables optimal nutrient utilization and enhances animal performance due to increased surface area allowing better contact with digestive enzymes. Moreover, adequate diminution of feed ingredients is beneficial to feed manufacturing processes such as mixing and hydrothermal treatments including pelleting, extrusion, and expansion. However, emerging trends in consumer and regulatory demands for restriction or cessation of animal production practices such as use of antimicrobial growth promoters are challenging current approaches to feed processing. There is limit as to the fineness of the particle size, as very fine particles negatively affect gut health due to higher incidences of stomach ulceration in pigs and gizzard dysfunction in poultry. Coarse particle size increases stomach and hindgut acidification which may be beneficial in controlling proliferation of enteric pathogens such as *salmonella* and *E. coli*. Optimal particle size could be designed in the grinding process using roller or hammer mill. However, since most commercial pigs and poultry diets are subjected to hydrothermal processes, additional reduction of feed particle size is inevitable. The need to achieve high physical quality and to reduce potential levels of feed-borne pathogens such as *Salmonella* has led to the application of relatively high conditioning temperatures during conventional hydrothermal processes, a practice that does not favor high nutrient utilization and stability of heat sensitive feed additives such as feed enzymes. Therefore, with evolving pig and poultry production practices, the regimens for feed processing will no longer be appreciated only in terms of optimizing nutrients utilization, but also in terms of impact on feed hygienic status, efficacy of feed additives, animal health, and food safety.

## Introduction

Advances in genetics has certainly produced commercial strains of poultry and pig with greater performance (growth, reproduction etc.) with minimal feed input. For example, over the last 5 decades, the body weight of broilers at 42 days has increased by 25–50 g per year and the feed conversion ratio to 2 kg body weight has improved 2–3 points annually (1, 2). A review of North Carolina white leg horns performance tests from 1958 to 2011 showed that the average age at 50% production decreased by 34 days, pullet body weight at point of lay dropped from 1.61 to 1.16 kg, mature hen body weight from 2.05 to 1.68 kg, feed conversion improved from 2.90 to 1.99, while egg mass increased from 16.3 to 19.9 kg per hen housed (3). With the introduction of crosses in the early 60's, specialization in dam and sire lines have been very successful in effecting genetic improvement of economically important traits in pigs, especially daily gain, backfat thickness, feed efficiency, and litter size. An annual genetic progress for gain of +20 g/day, lean meat of +0.5% and litter size of +0.2 piglet/litter has been achieved over the last few decades (4, 5). The nutrition of these animals has also evolved overtime but not as much as genetic advances; for example genetic selection brought about by breeding companies is responsible for 85–90% of the improvements in broiler growth, and advances in nutritional management contributed only 10–15% (1). However, the necessity to achieve and sustain genetic potential has been the driving force behind continuous advances in nutrition concepts seen in modern day commercial pig and poultry enterprises. In this context, feeding, a major control point of profitability has evolved and progressed both in terms of understanding digestive physiology and metabolism, and in the more precise evaluation of the quality of dietary raw materials. Advances in monogastric nutrition is clearly exemplified by the widespread adoption of net energy, standardized ileal digestible amino acids ideal ratio and digestible phosphorous concepts enabling nutritionists to formulate cost-effective and optimal diets (6, 7). Application of these concepts have also stimulated tremendous investments in commercial research and development in speciality feed ingredients such as crystalline amino acids and feed additive technologies such as feed enzymes, probiotics, and organic acids among others to further optimize nutrition (8, 9). Feedstuffs processing and diet manufacturing have also evolved such that the composition, ingredient choices, and diet form have been refined to improve feed intake and efficiency. However, the modern-day nutritionists perceive dysfunctional gastrointestinal tract as a potential rate-limiting factor in the survival and productivity of monogastric farm animals. This perception has been fostered by the emergence of ideas and concepts concerning the development and function of the digestive tract in the light of advances in genetic improvement and restriction on the use of antibiotic growth promoters and anti-coccidial drugs. The intention of this chapter is to provide a critical overview of feed processing with emphasis on particle size and hydrothermal processing (HTP) in the context of gut health and function. Implication of feed processing on application of exogenous feed enzymes will also be discussed.

## Feed Processing

The principal role of feedstuffs is to provide nutrients that can be digested and utilized for maintenance and productive functions. To maximize performance, pig and poultry diets must contain the correct balance of the essential nutrients required to meet the nutritional needs of various stages of production (6, 7). However, applying accuracy and a degree of precision in diet formulation requires an intimate knowledge of the animal, its daily nutrient requirements, feed intake potential and a more comprehensive understanding of the ability of the selected feedstuffs to provide target nutrient at least/best cost (10, 11). The range of feedstuffs incorporated into modern monogastric diets is continually changing due to several factors such as price volatility, component pricing dynamics, emerging novel, and opportunity feedstuffs, government regulatory regimens among many other reasons (12–20). However, feed processing must constantly produce feed products that are palatable, safe, and meets nutritional needs of the target animals. In this context, feed technology has progressed from simple mixing of mash feed to advanced preparations that involves various physical and hydrothermal processing operations (21). Today, most pig and poultry feeds are manufactured by employing a combination of technologies including physical grinding with hammer and/or roller mills in conjunction with hydrothermal processing including pelleting, expansion, or extrusion (21, 22). Indeed, feed processing includes single or multiple manipulation of feedstuffs or complete feed prior to presentation to the animal (22). Many advantages that can be attributed to feed processing includes improved availability of nutrients, destruction of inhibitors and toxins, facilitation of the use of a wide range of raw materials in diet formulations, production of hygienic feed, and reduction of feed wastage (21, 23). However, it is well-recognized that processing parameters such as extent of particle modification, processing temperature, pressure, duration, and water determine the physical and chemical reactions in and between nutrients as well as the adhesive properties on the feed particle surfaces, the final physicochemical structure and the hygiene status of the feed (22, 24). These attributes can directly and indirectly influence the impact of the processed feed on the digestive tract ecology and thus animal health, performance, and feed cost. There is a large body of reviews on aspects of feed processing in terms engineering (21, 24–26) as well as animal performance and feed economics (23, 27, 28). Subsequent sections will focus on the impact of particle size and HTP on gut health and function.

## Particle Size

Pigs and poultry are simple stomached animals largely dependent on repertoire of endogenous enzymes for their nourishment. One of the most important factor that determines feed utilization in these animals is the particle size distribution. Cereal grains are primary energy sources in monogastric diet and they require to be processed before or after mixing with other diet components. Particle size reduction always includes grinding step with hammer or roller mill to facilitate further processing (e.g., mixing, pelleting, extrusion, expansion). There are numerous reviews on the benefits of grinding feed ingredients in terms of milling throughput, nutrient utilization, growth performance and economics (22, 27–29). With respect to animal performance, the smaller the particle size the greater is the feed utilization because of increased specific surface of feed particles allowing better contact with digestive enzymes. The quality of grinding is assessed by factors such as homogeneity, uniformity, and size of the feed particles. One of the main challenges in monogastric feed manufacturing is uniformity and mixing homogeneity i.e., particle size distribution (22, 28). The feed industry strives to produce homogeneous feed, however, it has been reported that different factors including particle size, particle shape, density, electrostatic charge, dustiness, hygroscopicity, and flowability can significantly affect the quality of the feed mixtures (21, 29, 30). Particle characteristics, particularly particle size, are one of the most controversial issues in pig and poultry nutrition. From economic point of view, optimal particle size distribution adapted to physiological needs of animal enables optimal utilization of nutrients and enhances animal performance. However, recommendations regarding optimum particle size is contradictory as the results from feeding trials are confounded by a number of factors including feed physical form, complexity of the diet, grain type, endosperm hardness, grinding method, pellet quality, and particle size distribution (27, 28). In general, it is recognized that finer grinding increases the energy consumption at the mill and decreases capacity of grinding equipment and flowability, increases dust problems, and most importantly, too fine particles are associated with negative impact on gastrointestinal tract health and function.

## Impact of Particle Size on Gut Physiology

### Pigs

Gastric ulcers are one of the most important causes of sudden death of market hogs and can result in large economic losses (31). Presentation of gastric ulcers is typically in non-glandular gastric mucosa (*pars esophagea*) and estimates indicate that 1–2% of growing-finishing pigs die from gastric ulcers annually (31, 32). The reasons for occurrence of gastric epithelial alterations have not been clearly elucidated but numerous reports indicates feed particle size of cereals and other feed components are risk factors (Table 1) (38, 40–43). The presence of high quantities of fine particles in pig feed lead to higher incidence of stomach ulceration and other negative alterations of gastric mucosa as exemplified by keratization and mucosal erosion (28). In this context, concept of optimal particle size of pig feed is a widely researched aspect. Finer particles tend to increase fluidity of the stomach content which is associated with lesions of the *pars esophagea*. Pigs fed a coarse diet have heavier stomachs than pigs fed a fine diet, which probably reflects that coarse diets require more muscular action for processing by the stomach than fine diets. However, deleterious effects of finer particle size in pigs is dependent on grain type. For example, macroscopic keratosis scores were greater for pigs fed 0.30 vs. 0. 90 mm corn and hard sorghum but lower for pigs fed 0.30 vs. 0.90 mm soft sorghum (38). The effects of feed particle size on small and large intestine is less clear than in the stomach. However, an increased crypt depth in the colon was observed in pigs fed coarse diets (44, 45). This was linked to increased flow of undigested starch in the hindgut promoting production of butyrate, a preferred substrate for the colonocytes.

**Table 1:** Impact of feed particle size on gastrointestinal physiology in pigs and poultry.

**Species, age/BW**	**Particle size range, cereal**	**Effects, larger vs. smaller**	**References**
**BROILER CHICKENS, AGE IN DAYS**			
21–42	0.34–1.12 mm, corn	Increased gizzard weight and duodenal VH and CD	(33)
1–21	0.84–1.16 mm, wheat	Increased, crop, gizzard, small intestine, and ceca weight	(34)
1–21	0.59–0.95 mm, corn	Increased gizzard weight, no effects on ceca weight	(35)
1–42	0.65–1.3 mm, corn	No effects on gizzard and small intestine weight	(36)
**LAYERS, AGE IN WEEKS**			
20	0.15–2.5 mm, corn, wheat	Increased gizzard and GIT weight. No effects on histomorhology	(37)
**PIGS, BODY WEIGHT RANGE, KG**			
5–18.0	0.30–0.90 mm, corn, hard sorghum	Reduced stomach ulcerations. No effects on intestinal histomorphology	(38)
5–18.0	0.30–0.90 mm, soft sorghum	Reduced stomach ulcerations and no effects on SI histomorphology	(38)
50–100	0.40–1.00 mm, corn	Reduced stomach keratosis	(39)
30–60	0.43–1.10 mm, barley	Reduced stomach ulcerations, no effect on SI histomorphology	(40)
60–90	0.40–1.30 mm, wheat	Reduced stomach ulceration	(41)
5–100	0.50–1.25 mm, corn	Reduced stomach ulcers	(42)

### Poultry

Proventriculus and gizzard are the true stomach compartments, HCl and pepsinogen are secreted in the proventriculus and mixed with contents in the gizzard via muscular movements. However, because poultry do not have teeth, the gizzard has an important additional function of grinding feed material. Peculiarity is that the gizzard contains strongly myolinated muscles and has a koilin layer that aid in the grinding process (46). Detailed overview of gizzard functionality and regulation has been described (46, 47). Experimentations indicate that proventriculus and gizzard should be considered as one compartment with respect to digestive function where material flows rather rapidly through the proventriculus but will potentially be refluxed back into the proventriculus repeatedly during gizzard contractions. Lack of structural component in poultry diets has been associated with dilated proventriculus and a non-functional gizzard consequently compromising feed utilization and intestinal health (46, 48, 49). It has been reported that the volume of the gizzard may increase substantially when structural components such as whole or coarsely ground cereals are added to the diet (Table 1) (33–37), sometimes increasing to more than double the original size (46). The peculiarity is that when the diet contains structural components, digestive function improves through increased retention time, lower pH, and better grinding. These mechanisms in conjunction with better synchronization of feed flow are thought to improve nutrient utilization (46). Nir et al. (50) reported that a greater coarseness of feed increased the relative gizzard weight, whereas Amerah et al. (51) suggested gizzard stimulation was due to the length of time that the coarse particles resided in it. However, the effect of feed form (discussed later) must be considered in combination with particle size. Interestingly, it has been reported that longer retention times of the digesta in a well-developed gizzard might modify dietary protein digestion dynamics through increased HCl and pepsin secretion (52). Because of gizzard grinding, particles reaching the small intestine have no relationship with feed particle size, therefore, the impact of feed particle size on small intestine and ceca physiology is minimal (27).

## Impact on Gut Microbial Activity

### Pigs

Feed particle size distribution has been associated with strong influence on the presence of enteric bacteria pathogens. Data indicate that coarse feed particle size decreases pH in the stomach content compared with fine particle size linked to changes in gastric physicochemical and microbial properties (44, 45). Mikkelsen et al. (44) showed that coarsely ground feed increased solid gastric content, anaerobic bacteria count and concentration of organic acids in pigs. Further *in vitro* experimentation with the stomach content of pigs fed coarsely ground feed showed increased death rate of *Salmonella enterica serovar Typhimurium* DT12. This was associated with significantly higher concentration of undissociated lactic acid as exemplified by a strong correlation between the concentration of undissociated lactic acid and the death rate of *S. enterica serovar Typhimurium* DT12. These data demonstrated that pigs fed coarsely ground feed had much higher gastric microbial fermentation than pigs fed finer diets linked to slower gastric passage rate, increased gastric dry matter content and consistency (44, 45, 53). Gastric acidification in suckling pigs is mainly due to the presence of lactic acid, resulting from bacterial fermentation of lactose (54, 55). Cranwell (56) demonstrated that piglets achieved maximal gastric HCl output at the age of 5–6 weeks and that exposure to solid feed was important in this process. It follows that at weaning, the piglet not only loses lactose induced acidity but the ensuing anorexia exacerbate the ability of physiologically immature gut to produce enough HCl to keep stomach pH at an optimum of 3.5 (8, 9). Furthermore, diets fed to young pigs often have a high buffering capacity, which can further reduce stomach acidity (57–59). At low gastric pH, digestion of protein and populations of beneficial bacteria (*lactobacilli*) are maximized and harmful bacteria such as enterotoxigenic *E. coli* are inhibited (8, 9, 58). Consequently, gastric conditions created by coarse feed are interpreted to create additional “barrier” against fecal/feed-oral pathogenic bacteria transmission. Moreover, lower pH in digesta matrix sustains a higher proportion of short chain fatty acids (SCFA) in undissociated form and therefore antimicrobial potency (57). It seems that weaned pigs can benefit tremendously from coarsely ground feed, however, there are apparently limited research investigating manipulation of weaned pig feed particle size to characterize impact on gut health and subsequent performance.

Feed particle size not only impacts gastric ecology but also other parts of the GIT particularly the large intestine. Studies have demonstrated that coarse diets were strongly associated with higher propionic and butyric acid levels in the cecum and colon contents (42, 44). It is possible that coarse feed particle size may promote an increase of bacteria populations producing SCFA and, thus, contribute to gastrointestinal health by preventing the proliferation and/or virulence of harmful bacteria such as *Salmonella* spp. and *E. coli*. Studies have demonstrated that change in feed presentation could be associated with microbiota modification (different composition and/or metabolic activities) in the GIT of pigs (28). As alluded to studies have indicated that larger particle size increases flow of starch in the large intestines and this has been shown to increase SCFA production limiting growth of coliforms and *Salmonella* (28, 44, 45). Phenomenon of retrograde movement has been demonstrated in pigs and poultry where anti-peristaltic low amplitude waves in the hindgut (cloaca and colon) result in movement of digesta back to the ceca and distal ileum (46). The risk of this phenomenon is potential contamination of small intestines with hindgut pathogens. Cappai et al. (60) hypothesized that diet form could prevent retrograded contamination of small intestine by stimulating efficient functioning of the ileocecal valve. Pigs were fed diets differing in grinding intensity (roller vs. hammer) and sieve sizes (1 vs. 6 mm). Coarse meal significantly increased thickness of ileal cecal valve which was interpreted to have potential of preventing of digesta backflow into foregut. According to literature data, decreasing the quantity of fine particles in pig feed is strongly recommended (7). Generally, based on existing literature the quantity of finer particles (<0.4 mm) should be as low as possible due to the negative effect on GIT health and the quantity of the coarsest fractions (>1.6 mm) should also be low due to decreased nutrient utilization whereas the share of medium-sized particles (>0.5 to <1.6 mm) considered optimal for pig's digestive system should be as high as possible (38, 39, 43).

### Poultry

As in pigs, functional gizzard in poultry has been regarded as an important barrier in preventing pathogenic bacteria from entering the distal intestinal tract (22, 46). As alluded to, a well-developed gizzard enhances the grinding action, generates stronger reverse peristalsis contractions, increases proteolysis, and stimulates secretion of HCl which reduces the pH. The feed pH is close to neutral, high feed intake orchestrated by HTP treatments such as pelleting (discussed later) results in elevated gizzard pH unless gastric juice secretion can increase in accordance with intake (46). Thus, the gizzard pH is reported to be higher in birds fed pelleted diets compared to birds fed mash diets linked to smaller particle size in pelleted feeds. Many experiments have demonstrated that when broilers are fed structural components in form of whole or coarsely ground cereals, or fiber materials, such as hulls or wood shavings, the pH of the gizzard content decreases by a magnitude of 0.2–1.2 units (27, 46). This has been associated with increased gizzard volume and longer retention time leading to higher HCl secretion (61). Harmful bacteria entering the intestinal tract via the feed have a greater chance of being suppressed in a highly acidic environment. Huang et al. (35) used the *S. enterica serovar Typhimurium* DT12 model developed by Mikkelsen et al. (44) to evaluate whether physical properties of feed influenced *Salmonella* colonization in broiler alimentary tract. Birds given fine particle size (0.3 mm) diet had a lower *S. enterica serovar Typhimurium* DT12 death rate compared with those receiving coarse particle size (0.9 mm) diet. A lower *S. enterica serovar Typhimurium* DT12 death rate in gizzard contents was associated with a relatively higher pH in the gizzard of birds fed fine particle diet.

There is dearth of data to support implications of changes in gizzard ecology on small intestine and ceca function and health. However, GIT ecology that favors growth of *Clostridium Perfringens* has been recognized as one of the key risk factors for the development of necrotic enteritis (NE); the most threatening disease in the broiler industry worldwide (62–64). The hallmark of this disease is the presence of typical necrotic lesions particularly in the mid-region of the GIT with detrimental effects on the digestive and absorptive capacity (64). An important factor worth considering with respect to NE is the role of *Eimeria* spp. the causative agent for coccidiosis. Coccidial infection damages the intestinal epithelium, allowing leakage of plasma proteins into the intestinal lumen—a rich nutrient substrate that *C. perfringens* can exploit for proliferation and toxin production (62). Therefore, GIT ecological conditions that prevent proliferation of *Clostridium Perfringens* and *Eimeria* are seen critical in controlling NE (62). Feed particle size may affect the physiological and morphological characteristics of the GIT and thus microbial status. Finely ground feed stimulated fast growth of *C. perfringens* than coarsely ground feed (65). Branton et al. (66) observed that birds fed coarsely ground wheat diet had 18.1% mortality due to NE, whereas birds fed finely ground wheat diet had 28.9% mortality. This was linked to course feed stimulation of gastric function, including secretion of HCl and better utilization of nutrients in the small intestines (65, 67). The peculiarity is that large flow of undigested protein and amino acids in the ceca results in production of unfavorable metabolites such as phenols, thiols, amines, ammonia, indoles that are toxic but most importantly increases the pH of the ceca content creating perfect conditions for proliferation of pathogenic bacteria such as *Clostridium* spp. (62, 63). Therefore, increased protein and amino digestion due to well-developed gizzard as result of coarse feed particle size can reduce pathogens in the lower GIT. However, studies examining interaction between experimental infection with *Eimeria* and whole wheat feeding in broilers have not been conclusive. Based on studies reviewed by Yegani and Korver (67), feeding whole wheat vs. finely ground wheat improved digestive tract function in healthy birds however responses in the context of *Eimeria* challenge were variable and ranged from no effects to exacerbation of infection. Because it is being increasingly recognized that poultry have a requirement for a certain degree of physical structure in their feed to meet their innate feeding behavior development, the inclusion of dietary structural components, such as coarse particles, insoluble fiber sources, and whole grains should be given consideration in the context of gut health in antibiotic and anti-coccidial free feeding programs.

## Hydrothermal Processing

Common hydrothermal processes (HTP) in feed manufacturing includes pelleting, extrusion and expansion. The principle behind these processes are agglomeration of small particles into larger ones by means of mechanical compression in combination with application of moisture, heat, shear forces, and steam pressure (21, 24). Pelleting is the most prevalent HTP method for manufacturing pigs and poultry diets. Currently, most of pigs and poultry feed are fed as pellets or crumbles. Offering feed in pellet or crumble form improves the economics of production by bettering feed efficiency and growth performance (22, 28). These improvements are attributed to decreased feed wastage, higher nutrient density, reduced selective feeding, increased starch gelatinization, improved palatability, decreased time and energy spent for eating, and more importantly increased feed consumption (22, 23, 28). The ingestion of optimal level of dietary nutrients is very much dependent on the level of feed intake. In the case of pigs and poultry in most commercial situations, *ad libitum* provision of feed is practiced, in which the animal is permitted to give expression to its appetite (or voluntary feed intake). However, the level of consumption observed in practical commercial situations is often lower than the potential feed intake due to physical or physiological constraints and/or negative interaction with environmental situations (68, 69). Therefore, feed processing regimen such as pelleting that stimulate feed intake is well-received by the industry. The pelleting process can also increase nutritive value of the diet. Increased energy utilization has been reported in pelleted compared with mash feed (28, 70, 71). It has been shown that broilers fed pellets have lower heat increment and utilize more of the feed energy for productive purposes than those fed mash (72). A primary reason for the increase in productivity has been linked to behavior, more specifically, reduced energy wastage due to less time eating and more time resting (70). Heat and moisture applied in HTP have also been shown to positively affect nutrient (starch, protein) digestibility depending on the ingredients (23). Volumetric density is also reduced in mash feed and this can impact the ability to consume sufficient nutrients for maximum production, particularly when diets are low in nutrient density (68, 69). Particle size in mash diets can further impact diet palatability and this effect is modified by pelleting (10). Regardless of the mechanism, pelleting diets affects the effective caloric value of feed. As energy is the most expensive component in monogastric diet, gaining extra calories by simply pelleting the diet is quite attractive to the industry. Indeed it has been suggested that the extra productive energy provided by pelleting can be favorably used as a non-nutritional factor by the feed industry to reduce dietary energy content (10).

There have been some informative reviews on aspects of HTP technologies for achieving end-product quality particularly the pellet quality (22, 24–26, 28). A major concern in the feed industry is that of ensuring food safety. There is a direct link between animal-feed quality and hygiene issues and the safety of human food of animal origin. It follows, therefore, that feed production and manufacture should be considered as an integral part of the food production chain (73), subject to quality assurance and food safety systems (74). Therefore, with evolving consumer demands and regulatory regimens, the quality of feed is no longer appreciated only in terms of supplying nutrients, but also in terms of hygienic status, direct effects on animal health and food safety. Understanding how feed manufacturing strategies affect bacteria inactivation in feedstuffs and/or gut microbial activity may become an important aspect of efficient animal production without antibiotics (22, 28, 75).

## Hydrothermal Processing and Microbial Status in the Feed and Gastrointestinal Ecology

### Impact on Feed Microbial Load

Currently, there are no regulations dictating techniques related to microbial control in feed processing. Consequently, feed manufacturing techniques differ based on throughput demands, geographical and climate restrictions, ambient conditions, diet formulation, ingredient availability, and various feed processing equipment (75). There are numerous studies indicating HTP significantly reduces microbial load in feed (22). Most *salmonella* and coliforms can be eliminated by pelleting at temperatures above 80°C, while spore-forming bacteria are resistant to pelleting process as high as 90°C (75–77). Heat resistance also varies among non-spore-forming bacteria. For example, *Salmonella typhimurium* was more resistant to pelleting at 82.2°C with 15% moisture than *Salmonella enteritidis* whereas *S. enteritidis* was more resistant to pelleting at 87.8°C with 15% moisture compared with *Salmonella haardt* (78). The main factors determining the efficacy of HTP on feed decontamination are temperature, processing time, pressure and moisture (22). It is important to note these data are specific to feed microbial levels during and immediately after manufacture and do not predict microbial levels post processing. It is well-known that hydrothermally processed feed is at risk of recontamination during the cooling process, transportation, delivery, storage in feed bins, and feedlines (79). The most crucial stage for recontamination of the processed feed is the cooling process since high volume of air traverses through coolers and dust collected from coolers might have a greater likelihood of contamination compared with the dust obtained from other areas (76). However, there are studies indicating that HTP reduces prevalence of *salmonella* in chickens (22). However, it is yet to be determined whether this can be maintained in commercial poultry and pig operations.

### Impact on Gut Physiology

It is well-known that dietary components *per se* (ingredients, nutrients and additives) can modulate development and functionality of the gastrointestinal tract including histomorphology, immune and endocrine systems as recently reviewed (80, 81). By modifying feed ingredients and feed presentation, feed processing will further impact these aspects as discussed below.

## Histomorphology

Hydrothermal processing further reduces feed particle size as exemplified by minimization of the differences in the particle size distribution of coarse and medium grindings (82). During pelleting process, the feed is passed through steam, which softens the feed particles before they are pressed through the die by the rolls in the pellet press, causing an additional grinding effect. Generally, there are limited studies on the impact of HTP on gut microstructure and morphology. The limited studies have concluded that HTP induced changes in the gut morphology and function cannot be separated from the effects on the microstructure and particle size of feed (34, 37, 61). As a consequence there are numerous studies that reported decreased gizzard and pancreas weights in birds fed HTP feed (Table 2) compared with mash feed linked to particle size reduction (22). The use of structural components, therefore, becomes even more critical in diets subjected to HTP. Numerous studies have shown that birds fed a pelleted diet had significantly decreased relative gizzard weight linked to the lack of stronger mechanical gizzard stimulation (33–37, 50). Comparative feeding of pelleted and mash feed in pigs showed that pelleting increased stomach ulceration linked to diminution of feed particle size during pelleting (39, 42, 43). It is thought that weaker mechanical stimulation by the feed might explain the higher pH found in the gizzards of pellet-fed birds due to a decrease in HCl secretion than in mash fed chicks (35). While HTP has a hypotrophic impact on the gizzard and sometimes in proventriculus, HTP can also affect intestinal morphology in poultry but published data does not give a clear picture regarding the trends and patterns (Table 2). However, regardless of diet form, the morphological changes observed in the distal part of the poultry gut could not just be because of particle size reduction by HTP. Such effects could be linked to changes in chemical characteristics of feed, nutrient bioavailability, digesta viscosity, microbial growth, and activity (22). For example, mash-fed hens had a higher glucose transport rate than hens fed expanded diets attributed to higher villus surface and increased expression of mucosal glucose transporters (37). Increased ceca weight in broilers fed pelleted feed relative to broilers fed mash-fed was linked to increased flow of undigested starch in the ceca leading to increased fermentation capacity (35). In a more recent study addition of oat hulls in pelleted wheat diet increased gizzard weight and holding capacity (83). Further studies are needed to determine the mechanism behind the stimulation effects of HTP on gut physiology, morphology and immunology.

**Table 2:** Impact of hydrothermal processing on gastrointestinal physiology in pigs and poultry.

**Species**	**Processing, main cereal**	**Effects, processed vs. mash**	**References**
**BROILER CHICKENS, AGE IN DAYS**			
21–42	Pelleting, corn	No effect on gizzard but increased duodenal villi height and crypt depth	(33)
1–21	Pelleting, corn	Reduced gizzard weight, but increased ceca weight	(35)
1–21	Pelleting, wheat	No effect on gizzard but increased duodenum and jejunum villi height and crypt depth	(34)
1–42	Pelleting, corn	Reduced gizzard weight but no effects on small intestine weight	(36)
1–21	Pelleting, wheat and sorghum	Reduced gizzard and small intestine weight	(50)
**LAYERS, AGE IN WEEKS**			
20	Expansion, corn and wheat	Reduced gizzard weight. Reduced duodenal villi height but increased ileal villi height	(37)
**PIGS, BODY WEIGHT RANGE, KG**			
50–100	Pelleting, corn	Increased stomach keratosis	(39, 43)
5–100	Pelleting, corn	Increased stomach ulcers	(42)

## Appetite Control and Neuromodulation

Among the nutrients in pig and poultry diets, starch is quantitatively the most important. Diets may contain up to 50% starch on a DM basis, and starch is the most important source of energy. In monogastric farm animals, enzymatically digestible vs. fermentable starch increases net portal glucose uptake and as a consequence increases energetic efficiency of starch use for protein and fat tissues accretion (84, 85). Therefore, large part feed processing focuses on optimizing starch gelatinization for increased glucose absorption in the small intestine (86). However, heat processing of feed ingredients may result in formation of resistant starch through retrogradation. Resistant starch is considered a functional component as it positively influences the functioning of the digestive tract, microbial flora, the blood cholesterol level, glycemic index and assists in the control of diabetes (87, 88). Heat processing of feedstuffs has been shown to influence kinetics of starch degradation in the digestive tract of pigs by shifting site and extent of digestion with implications on voluntary food intake and adiposity (89, 90). Studies in rodents have provided evidence that fermentation of resistant starch is an important mechanism for increased endogenous secretion of the gut hormones glucagon-like peptide 1 (GLP-1) and peptide YY; satiety-stimulating hormones that are released mainly in the ileum and colon (91, 92). This in turn influences insulin release when GLP-1 binds to receptors on pancreatic β cells. The general concept is that the resistant starch escapes to the large intestine impacting luminal microbiota composition, luminal SCFA concentrations, and the expression of host genes involved in SCFA uptake, SCFA signaling, and satiety regulation (92). The mechanisms relating to starch chemistry upon processing, SCFA production and endocrine responses requires a better understanding to optimize glucose homeostasis. Moreover, understanding the mechanisms involved in the complex interactions between the diet, intestinal microbiota, and intestinal tissue can assist in supporting GIT function and health via targeted modifications of the diet. Recent data in pigs indicated that molecular and morpho-function of mandibular gland of pigs may be influenced by physical form of diet. A coarser diet was shown to increase the expression of leptin and its receptor in the epithelial cells of striated ducts in growing pigs (93). Further studies in piglets demonstrated differential expression and localization of cannabinoid receptors type 1 (CB1) and cannabinoid receptors type 2 (CB2) in the mandibular glands in response to variable chewing activity due to different diets form (94). The authors opined that these findings suggested a link between the diet form and the functional molecules involved in appetite regulation.

### Impact on Microbial Activity

The diversity of the microbiota in a gut section reflects in part the types of nutrient substrates in that section. Gastrointestinal microbiota derives most of their carbon and energy from luminal compounds (dietary and/or endogenous) which are either resistant to attack by digestive fluids or absorbed so slowly by the host that bacteria can successfully compete for them (8). Since bacterial species differ in their substrate preferences and growth requirements, the chemical composition and structure of the digesta largely determines the species distribution of the bacterial community in the GIT. Consequently, bacterial community structure and metabolic function is very much dependent on digesta biochemical conditions, because of feed composition and attendant host physiological responses such as endogenous secretions. It is inevitable that the use of any feed processing technology that influences the digestibility of the diet will change the selection pressures on the resident microbiota which in turn will moderate the efficiency with which the host utilizes its feed (8). As alluded to HTP improves digestibility of nutrients and thus likely alter gut ecology. Generally, a large part of starch in feed ingredients is digested in the small intestine of pigs and poultry. However, especially in heat-processed ingredients, a fraction of the starch may be retrograded and designated as resistant starch. The latter fraction cannot, by definition, be enzymatically degraded in the small intestine by host enzymes and passes to the large intestine where it can be fermented by residing microbiota. There are numerous studies demonstrating that resistant starch modulated intestinal microbiota and increased the expression of genes responsible for gut development through the production of SCFA creating acidic and hostile environment for pathogen overgrowth (95). Moreover, there are numerous reports indicating that HTP changes physical chemical property of dietary fiber through increased solubility and particle size reduction (96). Acid extrusion (incubation in acids followed by extrusion) fiber rich corn distiller's grains with solubles facilitated more rapid degradation of non-starch polysaccharides and shifted fermentation to more proximal gastrointestinal segments (97). However, there are limited studies on the effect of HTP of feed on the bacterial composition and activity in the gastrointestinal tract of poultry and pigs.

### Pigs

Heat treatment of cereals for piglets (corn and barley) and steam pelleting increased post-weaning growth performance and changed fermentation profiles in the hindgut indicating that the microbiota composition or their fermentation capacity had changed (98). Investigations on the impact of mash and pelleted diets on adhesion of *Salmonella enterica serovar Typhimurium* DT12 to pig ileum showed that mash diets were better in protecting than pelleted diets (45). The authors explained that pelleted diets stimulated secretion of mucins that facilitated *Salmonella* colonization. Total *E. coli* load was markedly lowered in both the caecal and colon contents of mash-fed pigs relative to pigs fed pelleted diets (42). Interestingly, cecal contents of pigs fed pelleted diet had higher content of genes for fimbriae F4 compared with cecal contents of pigs fed mash diet (42). These fimbriae are important virulence factor that facilitate enterotoxigenic *E. coli* binding to the specific receptors on intestinal epithelial cells resulting in colonization and subsequently in the secretion of enterotoxins such as STa, STb, and LT leading to diarrhea in piglets (8, 55). Enterotoxigenic *E. coli* (ETEC) strains causing diarrhea are more often detected in neonatal and newly weaned pigs (55). Thus, reducing the prevalence and the persistence of ETEC in pig herds may contribute to protecting pigs from contamination between production cycles and to reducing the risk of cross-contamination of piglets in the production system. It would be interesting to test feed texture in animals experimentally infected with ETEC to better understand the mechanism involved and record degree of diarrhea mitigation.

### Poultry

*In vitro* simulation studies of gizzard contents of birds fed pelleted diets showed lower *Salmonella enterica serovar Typhimurium* DT12 death rate compared to gizzard content of birds fed mash diet (35). However, *in vivo* experiment showed that birds fed pelleted diets had significantly higher concentrations of *Salmonella enterica serovar Typhimurium* DT12 in the GIT than did mash-fed birds (35). Bjerrum et al. (99) reported that birds fed pelleted feed had higher numbers of *Salmonella* in gizzards compared with those given whole wheat. Interestingly, pelleted diets have been shown to increase concentrations of SCFA in the gizzard compared with mash feeds. However, the increased SCFA in gizzard was not accompanied with lower pH in gizzard of birds fed pelleted diet (35). Feeding pelleted diets increased ceca concentration of SCFA which was accompanied with decreased pH (35, 65). This was explained to be related to the fact that pelleting induced substantial reduction in particle size such that nutrients that entered the cecum were easily available for microbial fermentation. In poultry, the ceca is the reservoir for *Salmonella* (100). It is therefore of interest that birds fed pelleted feed had higher concentration of *Salmonella* than did mash-fed birds (35). It appears that the reduction of ceca pH orchestrated by increased concentration of SCFA in broilers fed pelleted diets was not effective in reducing *Salmonella* colonization (35). Markedly increased concentrations of *Salmonella* in the ceca of pellet-fed birds demonstrated that the gizzard pH orchestrated by increased HCl production in relation to feed structure might be a better strategy of reducing the ceca concentration of *Salmonella*. Indeed, studies have demonstrated that pelleting of feed increased the incidence of *Salmonella* in the contents of gizzards and ceca of growing broilers providing evidence that the gizzard may be an important critical control point for reducing *Salmonella* contamination in growing broilers (22). Increasing processing temperature led to an increase of *lactobacilli* in the crop and ileum, whereas *clostridia* and *enterobacteria* seemed unaffected by HTP (101). The impact of different HTP treatments in the crop and small intestine were mostly confined to *lactobacilli* and lactic acid concentration (101). This study concluded that typical HTP applied in feed does not significantly influence GIT microbial dynamics in poultry. Although the number of studies investigating the effects of HTP GIT microbiology of poultry are limited, a better understanding of the effects of steam conditioning time and temperature manipulations could help producers maintain hygienic, physical, and nutritional quality of feed in antibiotic free feeding programs.

## Feed Processing and Efficacy of Exogenous Feed Enzymes

Although pigs and poultry are highly efficient in converting feed to food products, they still excrete significant amounts of undigested nutrients. For example, broilers lose almost 25–30% of ingested dry matter, 20–25% of gross energy, 30–50% of nitrogen, and 45–55% of phosphorus intake in the manure (10). Pigs of different breeds and ages were observed to digest 78% of gross energy in typical corn and soybean meal diet (102). Addition of 30% corn dried distiller's grains with solubles to this diet resulted in further reduction of digestible gross energy. The undigested nutrients are excreted in the manure with negative implications on production efficiency, profitability and sustainability of farm operations. The peculiarity is that feedstuffs contains anti-nutritional factors (ANF) such as phytic acid or fractions that are not degraded sufficiently or indeed at all by the conditions and the array of digestive enzymes in the gastrointestinal tract (8, 9). This inherent digestive inefficiency in monogastric animals is seen as the reason of commercial development and application of exogenous feed enzymes technology. Indeed, amongst biotechnological feed additives, feed enzymes have made the most progress and impact in the feed industry over the last three decades (8, 9). As such the utility of feed enzymes in terms of nutrition and gut health and function are widely researched (8, 103–105). However, exogenous enzymes added to the diet must exert their effect during the short time from when the feed is moistened in the anterior digestive tract to the point that feed residues have passed the small intestine (46). Furthermore, the enzyme must be able to withstand the rigors of feed processing and digestive processes such as pH and endogenous proteases. This complicated matrix of conditions has been partly associated with the variation in the efficacy of exogenous feed enzymes (9, 11).

Moderate HTP temperature (65–85°C) improves availability of nutrients due to gelatinization of starch, rupture of the cell wall matrix and deactivation of enzyme inhibitors present in cereals (106). However, there is a wide range of temperature and time combinations used in the commercial feed manufacturing. As protein, exogenous feed enzymes are susceptible to hydrothermal denaturation, early studies indicated that the magnitude of enzyme inactivation increased with conditioning temperature and time (107). The advances in technology over the last two decades have addressed the challenge of feed enzymes thermostability through strategies such as post-pelleting spraying, granulation with hydrophobic materials and molecular engineering approaches to bolster intrinsic thermostability (11). However, the susceptibility of exogenous enzymes to HTP, regardless of the production and applied protection technologies is different. For example, effects of different pelleting temperatures 60, 70, 80, 90, and 100°C on the activity of fungal amylase and bacterial amylase added in barley, wheat and soybean diet suggested that fungal amylase can be pelleted at temperatures of up to at least 80°C and bacterial amylase up to 90°C without a considerable loss in analyzed activity (108). More than 65% of activity of a blend of cellulase, β-glucanase, and xylanase was lost in a barley, corn, dried grass, wheat bran, peanut meal and soybean meal diet subjected to extrusion (109). However, and surprisingly, the enzyme treated diet still improved the energy and fat utilization in laying hens compared with the control. In a second experiment in the same study, 52% of the blend activity was lost after pelleting, however, enzyme improved nutrient utilization in unprocessed and pelleted diet to the same extent (109). Such observations might suggest the residual activity was still efficacious post-processing or initial enzyme dosing was excessive of available substrates. Moreover, it has been demonstrated that effects of exogenous enzyme was more pronounced in diets subjected to HTP (110). Peculiarity is that HTP changes the structure of non-starch polysaccharides (NSP) by increasing the ratio of soluble to insoluble fractions (106). Under such circumstances the magnitude of enzyme response will be greater to counteract deleterious effects of solubilized NSP. It has also been speculated that high conditioning temperature may destroy cell walls, releasing substrates that would otherwise not be accessible to the exogenous enzyme (111).

Most exogenous feed enzymes have an optimum pH of between 4 and 6, but great variation may exist between different sources of enzymes, which results in a spectrum of catalytic activity between lower and higher pH (112). Therefore, it is essential to understand these digestive conditions and how they may vary to predict efficacy of exogenous enzymes. It is obvious that functionality of the stomach may have a large effect on responses to enzyme supplementation. Intermittent feeding will increase retention time and decrease pH of the crop, and structural components will increase retention time and decrease pH in the gizzard, as discussed previously. Supplemental phytase was able to degrade 50% of the phytic acid during 100 min of retention in the crop of broiler chickens (113). Despite this, an experiment designed to increase retention time in the crop and gizzard failed to demonstrate any improved efficacy of phytase (114). Functionality of the posterior digestive tract may also be affected by functionality of the gizzard due to structural components. A dysfunctional gizzard may allow too much and poorly degraded nutrients to be passed to lower gut. The implication of such eventuality may be morphological and microbiological changes in the lower gut and possibly affect efficacy of feed enzymes. Taken together, it appears that there are several fundamental mechanisms that may underlie a wide range of situations in which interactions between feed processing *per se* and feed enzymes application may occur. An understanding of these mechanisms may provide an opportunity to develop strategies for application of feed enzymes and other heat sensitive feed additives when added in feeds subjected to diverse processing regimens.

## Future Perspectives; TOWARD Optimal Feed Processing

The benefits of feed processing in terms of animal performance and economics are not questionable. Concerns pertaining to aspects such as pellet quality, nutrient digestibility, protein denaturation and milling efficiency will continue to stimulate innovations in feed manufacturing. However, advances in feed processing optimization will be challenged by emerging consumer and regulatory trends for restriction or cessation of production practices such as use of antimicrobial growth promoters. For example, feed processing should take consideration of increasing focus on dietary approaches (ingredients and physical characteristics) for maintaining healthy and functional gastrointestinal tract. Clearly coarse particle size stimulates stomach development and functionality. A dysfunctional stomach may allow too much and poorly degraded nutrients to be passed through, and thus an increased level of undigested nutrients may enter the ileum and ceca. This may lead to morphological and microbiological changes, however, there is dearth of data to support implications of such changes on gut function and health. Optimal particle size could be designed in the grinding process using roller or hammer mill. However, since most pigs and poultry are fed diets subjected to hydrothermal processing, additional reduction of feed particle size is inevitable. Because fine grinding is generally favored for high pellet quality, and because it is difficult to avoid further reduction in feed particle size during the pelleting process, fine particle size is almost inevitable in pelleted feeds. The possibilities to decrease the intensity of grinding of particles during pelleting, by variation of parameters of pelleting process, are very limited. Modified extrusion process (i.e., processing using expander) followed by shaping element as applied in pet industry could be alternative for pelleting to preserve particle size, however there is dearth of data to application of this approach in pigs and poultry feed manufacturing. Strategies such as addition of concentrated fibrous material may be more applicable in pelleted feed, but data is largely lacking as to applicability in practical diets. The need to achieve high physical quality and to reduce potential levels of feed-borne pathogens such as *Salmonella* has led to the application of relatively high conditioning temperatures during conventional pelleting processes, a practice that does not favor high nutrient utilization. However, the true impact of high conditioning temperatures application on nutrient utilization of pelleted diets has been neglected due to focus on physical pellet quality and feed safety. Further research is warranted to identify and evaluate other possible approaches to manufacture high-quality pellets at low conditioning temperatures. Advances in enzyme technology will continue and one can expect that better forms of enzymes will be developed in the future. The “next-generation” enzymes will be close to being “perfect,” with rapid and high specific catalytic activity (per unit of protein), good thermostability, high activity under a wide range of gut pH, resistance to proteolysis and good stability under ambient temperatures. Therefore, with evolving pig and poultry production practices, the regimens for feed processing will no longer be appreciated only in terms of optimizing nutrients utilization, but also in terms of impact on feed hygienic status, efficacy of feed additives, animal health and food safety.

## Author Contributions

AM is a graduate student of EK, she gathered some of the research articles used in the review and write-up related to feed structure in poultry. EK searched literature and wrote significant portion of the review and had overall conceptual and editorial responsibility.

### Conflict of Interest Statement

The authors declare that the research was conducted in the absence of any commercial or financial relationships that could be construed as a potential conflict of interest.
